# Neuroinflammation and schizophrenia – is there a link?

**DOI:** 10.3389/fpsyt.2024.1356975

**Published:** 2024-02-08

**Authors:** Cristiano Chaves, Serdar M. Dursun, Massimo Tusconi, Jaime E. C. Hallak

**Affiliations:** ^1^ NeuroMood Lab, School of Medicine and Kingston Health Sciences Center (KHSC), Department of Psychiatry, Queen’s University, Kingston, ON, Canada; ^2^ Department of Neuroscience and Behavior, Ribeirão Preto Medical School, University of São Paulo, São Paulo, Brazil; ^3^ National Institute for Translational Medicine (INCT-TM), CNPq, São Paulo, Brazil; ^4^ Department of Psychiatry (Neurochemical Research Unit) and Neuroscience and Mental Health Institute, University of Alberta, Edmonton, AB, Canada; ^5^ Section of Psychiatry, Department of Medical Sciences and Public Health, University of Cagliari, Cagliari, Italy

**Keywords:** inflammation, schizophrenia, biomarkers, psychiatric genetics, neurobiology, neuroinflammation, psychotic disorders

## 1 Introduction: a historical prelude to modern understanding

Hippocrates, often regarded as the founding figure in empirical medicine, was the first to acknowledge the impact of environmental factors on general and mental health in his work “In airs, waters, and places.” However, it took over two millennia for substantial epidemiological studies to explore the relationship between mental disorders, such as schizophrenia, and environmental factors like birth seasonality. Although Tramer’s groundbreaking 1929 study identified a pattern of winter-spring births in 3100 patients with psychosis, a consistent association with schizophrenia was established only by the end of 20th century (1).

## 2 Maternal immune activation (MIA) and increased relative risk of schizophrenia in offspring

Extensive epidemiological studies in the last two decades have consistently shown that exposure to prenatal MIA significantly increases the odds of developing schizophrenia later in life (2, 3). Khandaker et al.’s (2013) systematic review highlighted a two- to fivefold heightened risk (3), while a more recent metanalysis by Zhou et al. (2021), encompassing 23 observational studies, identified a more modest yet steady increase in psychosis risk among children born to mothers who experienced infections during pregnancy (OR = 1.25, 95% confidence interval (CI): 1.1-1.41; p = 0.001) (4).

It is hypothesized that inflammatory molecules, such as cytokines and chemokines (e.g., TNF-α, IL-1β, IL-6), triggered by prenatal pathogenic exposure, may penetrate the placenta, impair fetal brain development, and cause lasting disturbances in neurodevelopmental trajectories (5, 6). Support for this theory comes from various animal studies examining the effects of MIA on prenatal neurodevelopment and subsequent outcomes (6–12). Behavioral anomalies, as well as structural and functional brain changes attributable to MIA, coincide with the developmental onset of schizophrenia symptoms (13). For instance, Crum et al. (2017) reported cortical thinning in rats following MIA, as evidenced by longitudinal in vivo MRI studies (14). Furthermore, Vasistha et al. (2020) found that MIA disrupted the development of specific subtypes of cortical GABAergic interneurons (15), and Dickerson et al. (2014) noted a decline in inhibitory markers like glutamic decarboxylase in the dorsal hippocampus and abnormal synchrony between the dorsal hippocampus and the medial prefrontal cortex (16).

Notably, these findings are not confined to rodent models. Hanson et al. (2022) reviewed the impact of MIA on the development of the prefrontal cortex in nonhuman primates, illustrating the wider applicability of these results (8). Additionally, Purves-Tyson et al. (2021) detected an upsurge in immune markers within the dopaminergic midbrain in both humans with schizophrenia and murine MIA models, suggesting a potential common pathway in the disease’s complex etiology (9).

## 3 The vulnerability-stress-inflammation model of schizophrenia

The vulnerability-stress-inflammation model of schizophrenia has emerged from major genetic and epidemiologic studies, diverging from the traditional neural diathesis-stress disease model initially posited by Zubin and Spring in 1977 (17). This contemporary model, as discussed by Howes, McCutcheon, and Stone (2015) and Muller et al. (2015) (18, 19), integrates inflammation as a central element, emphasizing the interplay of inflammation and immune dysregulation with genetic and environmental factors (20, 21).

Inflammation and stress during critical developmental stages, such as the second trimester or early childhood, are significantly implicated in increasing the risk of schizophrenia (7). Studies consistently indicate that stress augments inflammation markers, reinforcing the connection between stress and inflammation (10, 19, 20). Moreover, childhood adversity is linked with changes in immune and inflammatory responses (22–26), which may also involve dysregulation of the hypothalamic-pituitary-adrenal (HPA) axis (27, 28). Giovanoli et al. (2013) showed that experiencing stress during puberty can expose hidden neurologic damage that results from immune activation before birth in mice (29).

Genetics also plays a crucial role in stress sensitivity, with certain variants linked to both inflammation and schizophrenia (30, 31). Steen and colleagues (2023) identified common genetic markers associated with both schizophrenia and systemic immune alterations (32). MIA can lead to methylation changes in schizophrenia-related genes, thus affecting susceptibility through genetic and environmental risk factors (33, 34).

Infections and immune dysregulation’s impact extends beyond early neurodevelopment. In a 30-year nationwide cohort of population-based register in Denmark, Benros et al. (2011) showed that autoimmune diseases combined with exposures to severe infections increased the risk of schizophrenia in a dose-response relationship: three or more infections and an autoimmune disease were associated with an incidence rate ratio of 3.40 (95% CI=2.91-3.94) (35). However, this association may be partly due to common genetic factors, particularly within the HLA region. This region, which is vital for immune system functioning, has been identified as having the most significant genetic link to schizophrenia (Benros & Mortensen, 2020) (36).

In a comprehensive meta-analysis of 145 studies, Romer et al. (2023) observed elevated levels of immunological markers in the cerebrospinal fluid (CSF) of individuals with psychotic disorders relative to healthy controls (37), corroborating the findings of Runge et al. (2021), who also reported significantly increased IL-8 concentrations in the CSF of patients with schizophrenia spectrum disorders (38). Additionally, a subsequent meta-analysis encompassing 86 studies by Clausen et al. (2024) further substantiated these findings by showing a widespread activation of the immune system in psychotic conditions, as evidenced by the heightened count of immune cells in both the blood and CSF of the patients assessed (39).

Halstead et al.’s (2023) meta-analysis also shows a consistent elevation in pro-inflammatory proteins, like IL-6, indicative of ongoing inflammatory protein alteration throughout the illness (40). Growing evidence indicates a genetic foundation for the association between cytokine levels and schizophrenia (41). Further extending this research, Gonzalez-Castro et al. (2024) conducted a meta-analysis involving around 5,000 patients with schizophrenia and discovered a significant correlation between genes regulating cytokines and the predisposition to the disorder (42).

The repercussions of immune dysregulation on neuronal signaling, synapse organization, and brain connectivity are also significant (19, 43, 44). Williams et al. (2022) identified a significant correlation between genetically determined IL-6 levels and gray matter volumes in the frontotemporal cortex of schizophrenia patients (45). Consequently, the review by Dwir et al. (2023) underscores redox and immune signaling as critical therapeutic targets in schizophrenia (46).

## 4 Expanding the two-hit model of schizophrenia: neuroprogression and alternative hypotheses

In the context of the vulnerability-stress-inflammation conceptual framework, schizophrenia is currently conceptualized as a neurodevelopmental disorder driven by a two-hit process. The first hit arises from MIA and increased inflammation during the fetal and perinatal stages. This initial hit primes the immune system, particularly impacting the microglia, the brain’s primary immune cells responsible for repairing damage and pruning neurons (5, 34, 43, 47).

Microglia, often analogized as the “gardeners of the brain,” play a crucial role in maintaining neural health. They have a lengthy lifespan of 4 years, but some cells persist for up to twenty years (10). When primed, these cells can react to stress factors by initiating exaggerated pro-inflammatory responses, adversely affecting brain development (10, 20, 48). Intriguily, post-mortem and *in-vitro* research has shown that microglia are more active in eliminating synapses in neural cultures of schizophrenia patients, suggesting a key role in synaptic pruning (49).

The second hit typically occurs in late adolescence and leads to a series of significant changes: dopaminergic dysregulation, increased synapse pruning, and the emergence of psychosis (21). This phase is marked by an increase in dopaminergic transmission, presumably stemming from presynaptic dysregulation and receptor number changes in schizophrenia patients (18, 50). Correspondingly, animal models using NMDAR antagonism and MIA have reported reductions in gray matter volumes (14).

While the two-hit hypothesis highlights the critical roles of both prenatal and postnatal factors in schizophrenia, it does not fully address the progressive deterioration observed in patients. One of the earliest and most prominent brain alterations in schizophrenia is the loss of cortical gray matter volume, particularly in the fronto-temporal regions. This loss tends to worsen as the illness progresses, leading to increased severity and poorer clinical outcomes (51, 52).

Furthermore, neuroimaging studies have established a connection between schizophrenia and significant brain changes, including cortical atrophy and ventricular enlargement, which persist for at least ten years following the initial onset of psychosis. Notably, the extent of cortical atrophy has a stronger correlation with the presence of negative symptoms and cognitive impairments rather than with psychotic symptoms (53, 54). However, the absence of classic neurodegenerative signs in post-mortem studies has previously led to schizophrenia being labeled “the graveyard of neuropathology” (55, 56). Actually, the cortical atrophy is attributed not to actual neuronal loss but rather to diminished synaptic connectivity on pyramidal neurons (57–63).

Several new hypotheses have been proposed to deepen our comprehension of schizophrenia. For instance, Falkai et al. (2023) suggest that diminished maturation of oligodendrocyte cells may contribute to frontotemporal dysconnectivity and subsequent cognitive deficits (64). Interestingly, Morris et al. (2022) investigates how the endocannabinoid system could be linked to the persistent elevation of nitro-oxidative stress and immune dysregulation (65). Additionally, the magnitude of immune, cardiometabolic, and endocrine alterations in schizophrenia suggests it is better understood as a systemic disorder (66).

## 5 Low-level inflammation and neuroprogression in schizophrenia

Recent findings indicate inflammation may contribute to brain alterations seen in schizophrenia. Elevated levels of pro-inflammatory markers, including cytokines, are present in the blood and CSF of individuals with first-episode psychosis (FEP) and chronic schizophrenia (21, 67). Such persistent, low-grade inflammation is associated with changes in both the gray and white matter (44, 52, 68, 69). Furthermore, a systematic review has found that high levels of C-reactive protein (CRP) and interleukin-6 (IL-6) are linked with worsened clinical outcomes, exacerbation of symptoms, and structural brain changes, specifically diminished hippocampal volume and cortical thickness (69).

Moreover, schizophrenia patients exhibiting inflammation have shown increased macrophage activity and altered gene expression patterns in the brain endothelial cells within the frontal cortex (70). In a post-mortem study, Zhang et al. (2016) also observed an association between elevated levels of cytokines and reduced cortical gray matter volumes (71).

In addition to these structural brain changes, recent cohort studies have provided promising evidence for a link between inflammation and the negative symptoms associated with schizophrenia (72, 73). Interestingly, Kerkova et al. (2023) observed that a history of childhood trauma significantly moderates the relationship between inflammation and cognitive functioning in FEP (74). Furthermore, the increased size of the choroid plexus volume in antipsychotic-naïve FEP lends additional support to the significant influence of neuroinflammation on cerebral impairment (75).

## 6 Potential biomarkers for schizophrenia diagnosis and treatment

Schizophrenia diagnosis relies principally on clinical observation; however, assessing symptoms alone is subjective and lacks specificity for accurate diagnosis. In contrast, biomarkers offer a promising complement that can further delineate the boundaries of the diverse biological processes collectively termed schizophrenia (52). Biomarker discovery could bring a revolution with early detection, precise diagnosis, and more effective, targeted treatments (52, 76).

For instance, increased IL-6 levels in blood and CSF fluid are associated with more severe symptoms and poorer treatment response in schizophrenia patients (77, 78). Further, recent advances have identified several promising neuroinflammation biomarkers, particularly cytokines like interleukin-1β and interleukin-6 (19, 77, 79).

A significant breakthrough is the direct identification of markers indicative of microglial activation, such as the expression of 18-kDa translocator protein (TPSO), enabling the *in vivo* examination of microglia activation and neuroinflammation via TPSO PET imaging in the brain (20, 43). Despite the caveat that TSPO is found in various types of brain cells, not just in microglia (20), a metanalysis by Marques et al. (2019) found a significant increase in TSPO binding potential in patients with schizophrenia compared to controls (80).

Additionally, inverse correlations between CRP levels and cognitive function in acute psychosis have been documented (81–85). Further, Fathian et al. (2019) noted that reductions in CRP serum levels during acute psychosis are predictive of cognitive improvement over a six-month period (86).

Moreover, while a meta-analysis by Pilinger et al. (2019) on peripheral immune parameters in psychosis showed elevated cytokines, it did not support an immune subgroup hypothesis after adjusting for confounders (87). Contrarily, Enrico et al. (2023) identified a subgroup of FEP patients with high expression of inflammatory and immune-activating genes (IL1B, CCR7, IL12A, and CXCR3), suggesting heterogeneity within the condition (88).

## 7 Research studies in the present topic

The current Frontiers in Psychiatry Research Topic, titled “Further Findings in the Role of Inflammation in the Etiology and Treatment of Schizophrenia,” includes four original research studies that use inflammatory biomarkers as informative tools for schizophrenia diagnosis and treatment.

### 7.1 Predicting schizophrenia treatment outcomes with immune and metabolic parameters

Low-grade inflammation, present in both FEP and chronic schizophrenia, can be detected in routine laboratory tests. Skalniak et al.’s study indicates that lower CRP and C4 complement levels predict greater symptom improvement after 12 weeks of antipsychotic treatment (89). In accordance, Dr Yolken’s team, with over two decades of research on infections and immune dysregulation in schizophrenia, recently published a pilot study discussing the potential value of CRP and C4 complement as informative biomarkers in schizophrenia (90).

### 7.2 Exploring cellular senescence-related genes in schizophrenia diagnosis and treatment

Mounting evidence suggests an accelerated aging process, known as senescence, in individuals with schizophrenia and severe mental illnesses (91). Shortened telomeres in white blood cells serve as a key indicator of cell senescence. Feng et al.‘s study employs genetic tools to explore novel treatments for schizophrenia, highlighting the immunoglobulin gene myosin light chain kinase (MYLK) as a potential drug target for mental health therapeutic intervention (92).

### 7.3 The impact of exercise on inflammatory markers in individuals with schizophrenia

Given the implication of chronic low-grade inflammation in schizophrenia, Bigseth et al.‘s study investigates the effects of high-intensity exercise on inflammatory markers in schizophrenia patients (93). Despite the 12-week exercise program having no reducing effect on inflammatory markers, this outcome aligns with a recently published study on the same sample. The earlier study demonstrated no improvement in schizophrenia symptoms; however, a notable positive correlation was observed with the amelioration of depressive symptoms (94).

### 7.4 Clozapine’s role in inflammation among outpatients with schizophrenia

A recent investigation by the FondaMental Expert Centers for Schizophrenia in France (FACE-SZ) revealed a potential immune signature associated with the IL-23/IL-17 pathway in treatment-resistant schizophrenia (95). Additionally, Cordova et al.‘s study explores the immune profile of clozapine by comparing users and non-users. The study observes a correlation between clozapine use and an increased systemic immune inflammation index (SII) (96). However, a comprehensive understanding requires longitudinal studies with larger sample sizes to thoroughly assess the intricate relationships between inflammatory markers, disease progression, and the response to clozapine.

## 8 Conclusion: the future of inflammation-based schizophrenia research

While these studies contribute valuable insights, ongoing research, collaboration, and advanced technologies are crucial for unraveling the complex role of immune dysregulation in schizophrenia pathophysiology. The identification of potential biomarkers related to inflammation opens new paths for precise diagnosis and tailored treatments, offering promise for individuals grappling with this challenging mental health disorder.

## Author contributions

CC: Conceptualization, Writing – original draft, Writing – review & editing. SD: Conceptualization, Writing – review & editing. MT: Conceptualization, Writing – review & editing. JH: Conceptualization, Writing – review & editing.
